# Techno-Hypochondria: A Concept Analysis of Wearable Technology-Induced Health Anxiety Among Healthcare Professionals—Implications for Nursing Management

**DOI:** 10.3390/healthcare14131971

**Published:** 2026-07-02

**Authors:** Serpil Celik Durmus

**Affiliations:** Nursing Management Department, Faculty of Health Sciences, Kırıkkale University, Kırıkkale 71450, Türkiye; serpildurmus@kku.edu.tr; Tel.: +90-0530-9334650

**Keywords:** nursing management, technohypochondria, wearable health technologies, concept analysis, patient safety, workforce management, digital health literacy

## Abstract

**Highlights:**

**What are the main findings?**

- Conceptual Definition: This study formally defines “Technohypochondria” through three core attributes: biometric data obsession, digital catastrophizing, and a pathological need for algorithmic feedback. Unlike cyberchondria, it is established as a unique dependency on real-time, personalized biometric data streams.
- Antecedents and Consequences: The analysis identifies wearable technology ownership and low digital health literacy as primary antecedents. Critically, this phenomenon leads to systemic consequences, including clinical distraction, increased risk of medical errors, and the erosion of professional nursing autonomy.

**What are the implications of the main findings?**

- Managerial and Institutional Shifts: The findings necessitate a strategic shift in nursing management by making the human-technology relationship a standard of occupational health, requiring managers to develop institutional policies for wearable device usage to mitigate digital distraction and ensure patient safety.
- Educational and Theoretical Advancements: Nursing curricula should be updated to integrate digital health literacy as a psychological coping skill, while theoretically, this study provides the essential framework for developing a “Technohypochondria Scale” to quantitatively measure its impact on workforce productivity and clinical outcomes.

**Abstract:**

**Background and Aim:** While the proliferation of digital health technologies and wearable devices provides nursing professionals with constant access to biometric data, the pathological reliance on these metrics represents an emerging, yet empirically unexamined, digital anxiety framework. This study aims to theoretically define and systematically analyze this theorized phenomenon—termed “Technohypochondria”—within the context of nursing management and clinical practice. **Methods:** Utilizing Walker and Avant’s eight-stage concept analysis method, a systematic literature search was conducted across PubMed, CINAHL, Scopus, and Web of Science databases. Following strict inclusion and exclusion criteria, a total of 1240 data sources spanning nursing, management, psychology, and informatics literature were analyzed. **Results:** Three defining attributes of Technohypochondria emerged inductively from the literature: (1) Biometric data obsession, (2) Digital misinterpretation and catastrophizing, and (3) Need for algorithmic feedback. Unlike the general informational search patterns of cyberchondria, these attributes specifically capture a continuous, device-driven feedback loop. Ownership of wearable technology and inadequate digital health literacy were identified as primary antecedents. The analysis revealed significant managerial consequences, including loss of clinical focus, increased risk of medical errors, and weakened professional autonomy. **Conclusions:** Technohypochondria operationalizes a specific anxiety framework driven by constant biometric monitoring, conceptually diverging from cyberchondria’s focus on online health-information seeking. For nursing managers, addressing the psychological relationship between staff and technology is a strategic necessity for patient safety and workforce productivity. A primary limitation of this study is its theoretical nature; however, this study provides the essential conceptual foundation awaiting future empirical validation and scale development.

## 1. Introduction

Digital transformation in healthcare systems has moved beyond institutional medical devices to widespread adoption of mobile and wearable technologies, enabling individuals to continuously monitor their health metrics. Today, nursing professionals and patients have 24/7 access to biometric data such as heart rate, sleep quality, and stress levels [1]. While these technologies offer significant opportunities for proactive health management and clinical research [2], their integration into the daily lives of clinical staff has introduced unprecedented occupational and psychological challenges.

The phenomenon of health-related anxiety stemming from self-tracking and personal quantification is well-documented in recent digital health literature. Personal quantification often introduces hidden psychological costs, transforming objective biometric tracking into a source of continuous anxiety rather than empowerment [3]. In clinical cohorts, such as atrial fibrillation patients, the use of smartwatches has been shown to paradoxically contribute to heightened health anxiety due to constant monitoring [4]. Despite growing scholarly attention toward utilizing wearables to objectively assess anxiety levels [5], healthcare practitioners’ subjective experiences with self-tracking data reveal a complex vulnerability to data-driven stress [6].

The concept of “cyberchondria,” which describes excessive and repetitive online information seeking [2,3], remains insufficient in explaining this unique state of anxiety triggered by the instantaneous data flow from wearable devices. While cyberchondria focuses on external information pollution, “Technohypochondria” represents the pathological and obsessive relationship an individual establishes with their own biometric data. However, traditional tools like the Cyberchondria Severity Scale (CSS) are fundamentally designed to measure internet search behaviors, making them methodologically inadequate for capturing anxiety induced by immediate, automated device alerts. Similarly, broader frameworks like “technostress” primarily address organizational core-system hassles and user resistance [7], while “digital health anxiety” generalises anxiety over digital health interventions [8]. None of these constructs isolate the distinct cognitive trap where a user misinterprets their own real-time physiological data without external informational searching.

To fill this theoretical gap, this study proposes “technohypochondria” as a distinct candidate concept. Distinct from cyberchondria, technohypochondria is operationalized as a pathological, device-driven feedback loop characterized by an individual’s obsessive relationship with, and immediate somatic misinterpretation of, real-time biometric data. Wearable technologies create a continuous self-monitoring loop that chronicizes anxiety, fueled by technical limitations of wearables such as false positives, artifact noises, and measurement errors [9]. Consequently, a longitudinal pathway is established where underlying health anxiety precipitates a compulsive reliance on digital biomarkers [8] transforming routine device data into a persistent source of psychological distress.

Particularly among healthcare professionals, the tendency to “script worst-case scenarios” fueled by clinical knowledge (Clinical Hyper-vigilance) increases the risk of interpreting every deviation in device data as a serious pathology (self-diagnosis). While an “abnormal” signal from a wearable device may be a source of vague concern for an ordinary user, for a nurse, this data can be “over-clinicalized” as a potential arrhythmia or a precursor to myocardial infarction. This process, manifesting as “anxiety born of knowledge,” creates a new layer of workload that undermines professional attention management capacity. Grounded in Cognitive Load Theory (CLT), this continuous influx of unprompted, non-contextualized biometric data exceeds the working memory capacity of the clinician, converting objective metrics into a perpetual source of distress [10], This continuous cognitive overload and attention fragmentation directly mirrors the institutional phenomenon of “alarm fatigue” [11], shifting it to a personal, pervasive level [12].

When evaluated through the lens of the Job Demands-Resources (JD-R) Model, this unrelenting exposure to digital biometric feedback functions as an intrusive cognitive job demand, accelerating psychological strain and emotional exhaustion among clinical staff [13]. Paradoxically, while the Technology Acceptance Model (TAM) [14]. elucidates why clinicians initially adopt wearables for proactive health optimization, the onset of Technohypochondria causes this utility-driven behavior to degenerate into a compulsive dependency. This algorithmic reliance directly compromises the core tenets of Self-Determination Theory (SDT)—specifically autonomy and perceived competence—as the practitioner’s professional self-efficacy becomes subservient to continuous, automated validation [15]. From a human factors and digital dependency perspective, this feedback mechanism closely mirrors behavioral addiction paradigms, wherein individuals become tethered to intermittent algorithmic reinforcements, thereby fracturing occupational focus and cognitive resources.

Given that wearable technologies are increasingly studied for detecting physical burnout in healthcare professionals [11], unaddressed technohypochondria among staff poses an operational risk, directly impacting attention management, reducing job engagement, and threatening patient safety and workforce productivity [7]. Therefore, addressing this psychological relationship between nursing staff and wearable technology is a strategic necessity for modern nursing management.

Consequently, systematically defining and establishing the conceptual boundaries of this emerging phenomenon is paramount for workforce planning, quality of care, and the design of a technology-compatible work environment. The aim of this study is to define the concept of “Technohypochondria,” a new phenomenon in nursing literature, using Walker and Avant’s (2019) method of concept analysis [16]. Through this approach, the study systematically delineates the concept’s antecedents, defining attributes, and managerial consequences, providing a rigorous theoretical foundation awaiting future empirical scale development.

### Research Questions

1. How is the concept of Technohypochondria characterized in the context of high-tech clinical environments, and what are its core defining attributes?
2. What are the specific antecedents that trigger Technohypochondria among healthcare professionals, and what are its potential consequences on nursing management and health service delivery?
3. How can Technohypochondria be theoretically and operationally delineated from related or overlapping constructs, such as cyberchondria and traditional illness anxiety disorder?

## 2. Materials and Methods

### 2.1. Study Design

The primary objective of this study is to introduce the concept of “technohypochondria”—developed within the framework of self-monitoring pressure and cognitive load associated with wearable technologies among healthcare professionals—into the nursing literature. To ensure a rigorous conceptual architecture, this study provides a theoretical analysis using Walker and Avant’s (2019) eight-stage concept analysis methodology [16]. In strict alignment with this methodological framework, the analysis is sequentially structured across all eight mandated phases: (1) selection of the concept, (2) determination of the aim of the analysis, (3) identification of all usages of the concept, (4) determination of defining attributes, (5) construction of a model case, (6) construction of borderline and contrary cases, (7) identification of antecedents and consequences, and (8) determination of proposed empirical referents. This methodology was selected to establish the theoretical foundation of technohypochondria—a phenomenon that currently lacks a standardized definition in the literature—within the contexts of nursing management and clinical practice, thereby providing an operational framework to guide future quantitative scale development.

Because this study relies exclusively on publicly available, peer-reviewed theoretical and empirical literature as data sources, institutional review board (IRB) approval or ethical clearance for human subject participation was not required. No human subjects or confidential patient records were involved in any stage of this conceptual synthesis.

### 2.2. Data Sources and Search Strategy

A comprehensive, systematic literature search was conducted covering literature published up to April 2026 using the PubMed, Web of Science, Scopus, and CINAHL databases. To capture the foundational evolution of health-related anxiety alongside the exponential surge in AI-driven health tools and modern biometrics, no restrictive historical start-date was applied to the initial query, ensuring that seminal baseline frameworks were integrated analytically.

To guarantee transparency and full reproducibility as expected in structural reviews, the exact Boolean search strings utilized across the target databases were operationalized as follows:

- PubMed/Medline: (“technohypochondria” OR “digital health anxiety” OR “biometric data obsession”) AND (“wearable technology” OR “wearable devices” OR “smartwatch”) AND (“health personnel” OR “nursing professionals” OR “healthcare workers”)
- Web of Science/Scopus/CINAHL: (“digital health anxiety” OR “biometric data obsession” OR “technohypochondria”) AND (“wearable technology” OR “wearable devices” OR “smartwatch”) AND (“nursing” OR “healthcare professionals” OR “nurses”)

Database filters were restricted to English-language and peer-reviewed documents, including empirical studies, systematic reviews, and theoretical discussion papers, while gray literature, conference abstracts, and editorials were filtered out.

The manuscript was originally drafted in Turkish and subsequently translated into English using the DeepL translation tool (DeepL SE, Cologne, Germany). Following the initial translation, the authors conducted a rigorous manual review and refinement of the text to ensure technical accuracy and linguistic flow within the context of nursing. The final version was cross-checked with academic nursing terminology to maintain conceptual integrity.

### 2.3. Inclusion and Exclusion Criteria

To ensure the clarity and external validity of the concept analysis, a strict, multi-stage screening strategy was implemented based on predefined criteria. Studies were explicitly included if they addressed: (1) cognitive, behavioral, or psychological responses to continuous physiological data tracking; (2) data-driven distress or technostress among adult cohorts; and (3) clinical workplace management implications of user-technology interactions. Papers focusing exclusively on undergraduate student populations were systematically excluded to ensure the concept’s manifestations were extracted from active clinical environments and heterogeneous professional frameworks.

To ensure the scientific credibility of the synthesized literature and mitigate biased extraction, all retrieved core sources underwent a rigorous quality appraisal using the modified framework by Whittemore and Knafl [17] evaluating papers based on methodological quality, data relevance, and conceptual contribution. Rather than using literature descriptively, data extraction was driven by a systematic thematic analysis procedure [18]. Two independent reviewers extracted data onto a standardized matrix to inductively map sentences, definitions, and findings that mapped onto the attributes, antecedents, and consequences of technohypochondria, resolving discrepancies through consensus.

A total of 35 key studies were included in the final analysis. This sample size was finalized upon reaching the point of “theoretical saturation,” where data regarding the attributes, antecedents, and consequences of the concept began to repeat, and no new thematic components emerged [16].

### 2.4. Literature Selection Process (PRISMA Flow Data):

**Figure 1:**
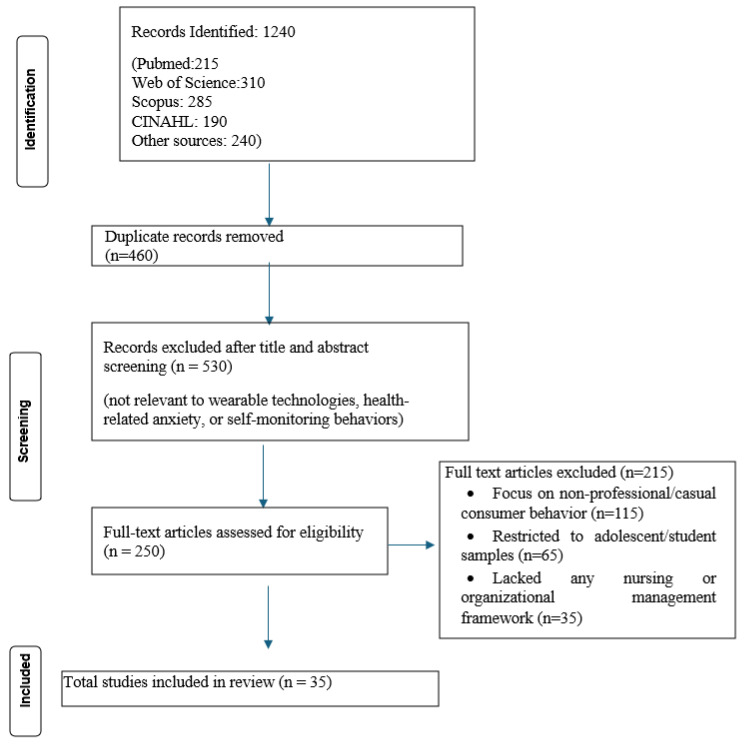
PRISMA—inspired flow diagram of study selection process.

The conceptual reflection in the literature was refined through the PRISMA 2020 guidelines for systematic reporting, mapping the explicit reduction of studies from identification to final inclusion (formally integrated as Figure 1 within the manuscript):

- Identification: Total records identified through systematic database searching: N = 1240 (PubMed: 215, Web of Science: 310, Scopus: 285, CINAHL: 190, Other sources via secondary citation tracking: 240).
- Screening: Following the automated and manual removal of duplicate records (n = 460), a total of 780 unique records were screened. During this phase, a title and abstract review led to the exclusion of n = 530 studies that did not directly investigate technology-induced health anxiety or biometric monitoring.
- Eligibility & Exclusion: Subsequently, n = 250 full-text articles were rigorously assessed for eligibility. Out of these, n = 215 papers were excluded based on clear justifications: n = 115 focused on non-professional/casual consumer behavior, n = 65 were restricted to adolescent/student samples, and n = 35 lacked any nursing or organizational management framework.
- Included: Consequently, a final sample of n = 35 core sources met all strict eligibility criteria to define the specific attributes, antecedents, and consequences of technohypochondria.

### 2.5. Methodological Quality and Critical Appraisal of Included Sources

To ensure the epistemological integrity and methodological rigor of the framework, all 35 included sources underwent a comprehensive methodological quality assessment utilizing the internationally validated Joanna Briggs Institute (JBI) Critical Appraisal Tools (specifically adapted for text/opinion papers, cohort studies, and qualitative designs as appropriate) [19]. The evaluation was independently conducted to assess internal validity, source credibility, and potential biases. Studies were scored based on the percentage of met criteria, categorized as high quality (≥80%), moderate quality (60–79%), or low quality (<60%). The appraisal demonstrated that 88.5% (n = 31) of the sources exhibited high methodological rigor, while 11.5% (n = 4) demonstrated moderate rigor, with no exclusions required due to low quality. The detailed item-by-item JBI appraisal scores for each of the 35 sources have been systematically integrated into the study as Supplementary Table S1.

## 3. Defining Attributes of the Concept of Techno-Hypochondria

According to the conceptual framework of Walker and Avant (2019), defining attributes represent the most characteristic features that establish the core identity of a concept and distinguish it from related or overlapping phenomena [16]. In the context of nursing management, clinical leadership, and healthcare informatics, the systematic synthesis of the literature reveals three distinct, interdependent defining attributes that characterize “Technohypochondria.”

### 3.1. Biometric Data Obsession

The foundational attribute of technohypochondria is a pathologically intensified focus on continuous, self-reported numerical data tracking. This baseline feature transcends the generalized web-based information-seeking behavior that typically defines cyberchondria. Instead, individuals exhibit a profound cognitive hypersensitivity toward real-time digital biomarkers—such as continuous heart rate, heart rate variability (HRV), micro-sleep stages, and blood oxygen saturation (SpO_2_)—generated by wearable biomedical devices, smartwatches, and rings.

Within this state, an individual reductionistically translates subjective bodily sensations into objective numerical outputs, establishing what the literature identifies as an absolute epistemic authority of the device over somatic reality [3,8]. These continuous feedback loops from wearable tools perpetuate an uninterrupted self-monitoring cycle, structurally chronicizing non-clinical anxiety [4,5].

Managerial Perspective: In nursing services management, this obsession shifts a professional’s cognitive resources away from critical patient surveillance toward personal biometric outputs. In high-acuity clinical environments, a nurse who prioritizes personal wearable alerts over objective patient monitors suffers severe “cognitive interference.” This attention fragmentation creates data-management vulnerabilities that directly jeopardize professional care continuity and compromise patient safety protocols.

### 3.2. Digital Misinterpretation and Catastrophizing

This attribute refers to the immediate cognitive distortion of non-pathological, instantaneous physiological fluctuations reported by a device, treating them as definitive evidence of an acute medical crisis. Rather than contextualizing digital metrics within normal baseline variations, the individual instinctively engages in cognitive catastrophizing, projecting worst-case clinical diagnoses [20].

Paradoxically, the advanced clinical knowledge and high health literacy possessed by nursing professionals do not mitigate this stress; instead, they act as an epistemological catalyst that accelerates the anxiety of potential misdiagnosis [21,22]. For example, a minor physiological tachycardia alert driven by routine ward ambulation is frequently over-clinicalized by an anxious practitioner as an impending acute cardiac event. This decontextualized interpretation strips data of its environmental reality, placing the professional inside a self-induced “digital panopticon” [9,23].

Managerial Perspective: Within clinical leadership frameworks, the anxiety generated by personally held, unverified biometric data begins to distort evidence-based decision-making. When a nurse’s professional autonomy is overshadowed by algorithmic anxiety, it induces a state of “computational paralysis,” significantly impairing executive functioning and critical interventions during emergency crises.

### 3.3. Constant Algorithmic Feedback Seeking

Technohypochondria is fundamentally sustained by an externalized dependency on algorithmic validation to authenticate subjective well-being. Individuals exhibiting this attribute systematically reject internal somatic signals of wellness unless they are verified by pre-calculated device metrics, such as “stress indexes” or “readiness scores” [24].

By delegating somatic self-awareness to automated digital algorithms, professionals reconstruct their physical experiences around commercial charts, graphs, and scores [6,25]. This transition seals a state of “digital health dependence,” where the practitioner becomes functionally incapable of perceiving themselves as fit for duty without receiving positive algorithmic confirmation from their wearable technology [26].

Managerial Perspective: This operational dependency severely erodes a nurse’s professional self-efficacy and clinical intuition. Pre-emptive cognitive framing—such as a nurse assuming, “My application reports poor recovery scores today, therefore my probability of clinical error is significantly elevated”—transforms algorithmic data into a negative self-fulfilling prophecy. Replacing professional clinical confidence with automated algorithmic validation poses a strategic human resources challenge, eroding workforce productivity, diminishing resilience, and undermining organizational commitment.

### 3.4. Data Synthesis and Inductive Thematic Coding Process

To ensure a strictly evidence-driven analysis and validate the empirical emergence of the construct, the 35 included sources underwent a rigorous, three-stage qualitative thematic coding process based on grounded theory principles. In the *first stage* (*Open Coding*), raw textual evidence regarding the psychological and behavioral responses to wearable data was extracted from the literature, yielding primary descriptive codes. In the *second stage* (*Axial Coding*), these initial codes were compared, systematically integrated, and categorized based on shared conceptual properties (such as biometric hypersensitivity and over-clinicalization). In the *third stage* (*Selective Coding*), these broader categories were systematically integrated to abstract the core defining attributes of the phenomenon. This systematic coding pipeline directly generated the framework presented in Table 1, mapping the traceable empirical pathway from raw literature findings to the operationalized dimensions of Techno-Hypochondria.

## 4. Cases

According to the Walker and Avant methodology, cases provide a concrete contextualization of the phenomenon under investigation by reflecting highly realistic representations of the core attributes in real-world scenarios [16]. Rather than relying on purely subjective intuition, the cases presented below are systematically synthesized from empirical clinical descriptions of technology-induced anxiety, wearable device dependencies, and workplace cognitive overloads documented in contemporary nursing management literature.

### 4.1. Model Case

Nurse Z is an experienced professional working in a tertiary intensive care unit, highly tech-savvy, and a consistent user of a smartwatch. During the peak and most stressful hours of the shift, the smartwatch sends a notification stating, ‘Your resting heart rate has risen above 110 bpm.’ Although this increase is a physiological response resulting from the immediate physical activity and workload, Nurse Z focuses intently on the device’s alert and begins to believe she has a latent arrhythmia or cardiac pathology (Biometric Data Obsession).

Instead of rationalizing the data from the device through her professional clinical knowledge, she succumbs to panic by considering the worst-case scenario and begins to manually check her pulse repeatedly (Digital Misinterpretation and Catastrophizing). Preoccupied with her ‘digital alarm’ and anxious about the drop in her watch’s ‘readiness score’ (Constant Algorithmic Feedback Seeking), she fails to promptly recognize the critical early warning signals emerging on the monitor of the patient under her responsibility. The condition of Nurse Z is not merely an external informational search (cyberchondria) [29]; it is a typical case of Technohypochondria, triggered by real-time biometric data from wearable technology, which directly impairs clinical performance and compromises patient safety.

### 4.2. Borderline Case

Borderline cases are examples that contain some of the attributes of the concept being examined but do not meet all the criteria, thereby serving to mathematically clarify conceptual boundaries [16].

Case Example: Nurse L, feeling fatigued due to the heavy workload in the ward, checks her smartwatch to monitor her heart rate. Upon seeing an elevated heart rate, she decides to take a brief 5-min break and subsequently returns to her normal working pace.

- Why is this NOT Technohypochondria? Although Nurse L uses biometric data for self-monitoring, she does not engage in catastrophizing or experience medical anxiety. Furthermore, the situation does not lead to a persistent impairment in her clinical performance or a dependence on algorithmic validation. In this instance, the device is utilized merely as a homeostatic ‘regulatory tool’ rather than a source of pathological concern.

### 4.3. Related Case

This is a condition that is closely related to the concept (such as cyberchondria) but remains distinct from it upon operational evaluation.

Case Example: Nurse M consistently searches for ‘brain tumor symptoms’ on internet forums and medical websites due to the headaches she has recently been experiencing. She continuously reads articles during her shift and feels anxious.

- Why is this NOT Technohypochondria? This is a classic case of ‘Cyberchondria.’ The source of anxiety is a generalized, static search for health information on the internet rather than real-time, continuous biometric data originating from a personalized wearable device.

### 4.4. Contrary Case

This represents the exact opposite of the concept, demonstrating complete conceptual divergence.

Nurse M is an experienced professional working in a tertiary intensive care unit who, similar to Nurse Z, is highly tech-savvy and a consistent user of an advanced smartwatch to track her daily health telemetry. During the peak, high-workload hours of a demanding shift, her smartwatch sends an identical automated notification stating, “Your resting heart rate has risen above 110 bpm.” Unlike the techno-hypochondriac profile, Nurse M immediately applies her professional clinical knowledge to rationalize the telemetry. Instead of focusing intently on the device’s alert or interpreting it as a latent cardiac pathology, she recognizes the spike as a normal, self-limiting physiological response to immediate physical exhaustion and occupational stress.

Furthermore, while reviewing her morning dashboard, she notes a significant drop in her watch’s “readiness score.” However, rather than succumbing to anxiety, catastrophizing the digital data, or engaging in obsessive manual pulse-checking, she views this score strictly as a supportive informational metric. She adjusts her physical pacing and performs brief deep-breathing exercises during a scheduled break without developing any algorithmic dependency. Most importantly, Nurse M maintains complete situational awareness: her interaction with the wearable device remains entirely non-pathological and functional, ensuring her primary focus remains directed at the critical early warning signals on her patient’s monitor. Her condition demonstrates that active usage of biometric wearables does not inherently lead to techno-hypochondria when mediated by objective cognitive filtering, thereby preserving clinical performance and patient safety.

- Why is this NOT Technohypochondria? Because the individual exhibits zero dependence on digital technology, preserves her internal somatic awareness, and completely maintains her professional clinical autonomy.

## 5. Identifying Antecedents and Consequences

In strict accordance with Step 7 of the Walker and Avant framework, antecedents and consequences must be rigorously derived from the existing peer-reviewed literature rather than speculative insights [16]. To achieve this, a systematic thematic extraction matrix was applied to the 35 analyzed sources, mapping out the precise chronological precursors and downstream operational impacts of the concept.

### Antecedents

The systematic literature synthesis indicates that the following preconditions must be present for the concept of Technohypochondria to manifest in an individual or a professional:

Ownership of Technological Hardware: A fundamental antecedent is the individual’s continuous access to wearable devices (smartwatches, rings, chest straps, etc.) capable of tracking biometric data and providing an uninterrupted, 24/7 data stream [5]. This technological infrastructure enables the individual to establish a constant “digital surveillance” over their own physiology [4,30].

Inadequate Digital Health Literacy: A critical antecedent is the lack of sufficient knowledge to distinguish the clinical significance and physiological variability of biometric data (HRV, tachycardia, stress scores, etc.), as well as the technical margins of error of the devices. The literature indicates that this gap between high data access and low interpretation capacity directly triggers health anxiety [21,22,31].

Individual Predisposition: The psychological foundation is formed by a high level of general anxiety, low tolerance for uncertainty, or a personality structure prone to hypochondriacal tendencies (illness anxiety disorder) [8,28]. This predisposition acts as a catalyst, causing even neutral data from the device to be encoded as “threat signals” [32,33].

## 6. Proposed Empirical Referents and Future Research Roadmap

Empirical referents, which define the measurability and field reflections of the concept, are of critical importance for detecting Technohypochondria in clinical settings and providing a foundation for future research. As a result of this analysis, parameters such as the frequency of checking personal health devices during working hours, anxiety scores observed following device notifications, and clinical decision errors resulting from the misinterpretation of digital data are proposed as fundamental metrics for evaluating the phenomenon of Technohypochondria [5,8].

Based on this analysis, four primary research strategies have been identified for future field studies. First, considering that existing cyberchondria scales remain insufficient in measuring wearable technology-focused biometric data tracking, a valid and reliable “Technohypochondria Scale” should be developed for nurses and patient populations [34,35]. To facilitate this operational development, a foundational structural roadmap identifying potential item domains and measurement markers is proposed in Table 2.

Second, there is a need for quantitative research examining the impact of Technohypochondria levels on nurses’ clinical focus levels and tendencies toward medical errors. In this context, the “mediator” role of Technohypochondria in the relationship between smart device use and technostress should be tested to clarify the impact of this psychological mechanism on professional performance [8,24].

As a third strategy, the effectiveness of structured training programs aimed at increasing the digital health literacy of nurses in reducing Technohypochondria-related anxiety should be demonstrated through quasi-experimental studies [21,22]. Finally, phenomenological interviews with healthcare professionals exhibiting these symptoms will provide an in-depth understanding of how individuals interpret data from wearable technologies and how this process shapes professional identity by creating a digital panopticon effect [25]. These empirical referents and research recommendations will ensure that the concept of Technohypochondria moves beyond theoretical debate and transforms into an evidence-based field of application for nursing management.

## 7. Discussion

This concept analysis conceptually outlines that the phenomenon of “Technohypochondria” in the rapidly digitalizing healthcare ecosystem is not merely an individual anxiety issue, but rather a complex phenomenon that transforms nursing management and clinical practice processes. Our conceptual assumptions, supported by current literature (2020–2026), indicate that the concept has evolved from cyberchondria and is constructed primarily upon “biometric data obsession.” To maintain a balanced perspective, it must be noted that for the vast majority of users, wearable health devices do not cause clinical impairment, but instead offer significant clinical utility by empowering individuals through early health indicators.

As a digital paradox, while continuous access to one’s own health data was initially viewed as a tool for empowerment, the present analysis suggests that this situation frequently transforms into a source of “digital uncertainty.” As emphasized by Lau et al. and Babu, monitoring biometric biomarkers without clinical guidance creates a technological obsession in individuals [8,21], although it should be critically noted that these foundational studies did not specifically examine healthcare professionals, making direct generalization a theoretical extrapolation. In this process, the individual rejects their own subjective bodily sensations and tethers their well-being solely to “algorithmic approval” [24]. This causes the principle of “somatic awareness,” which is of critical importance in nursing care, to be potentially attenuated by “dependence on digital data.”

To clarify the conceptual boundaries of this phenomenon, a crucial distinction must be made between a rational response to device alerts and a truly pathological reaction. As Rosman et al. described, smartwatch-induced anxiety in certain patient populations can represent an appropriate health concern aimed at detecting true physiological anomalies (such as atrial fibrillation) [4]. Technohypochondria diverges from this rational utility when the behavioral response transitions into a persistent, ungrounded cognitive paralysis that compromises operational focus. Furthermore, this construct must be carefully distinguished from related phenomena to avoid category errors, as systematically delineated in Table 3.

Beyond mere differentiation, examining the shared etiological pathways and the precise transitional mechanics between these constructs yields critical clinical insights. Cyberchondria and techno-hypochondria operate on a shared cognitive vulnerability—namely, an intolerance of uncertainty and a propensity for somatic catastrophizing [28]. However, when an individual with pre-existing cyberchondria adopts real-time biometric wearables, a profound clinical transition occurs. Cyberchondria relies on an active, episodic, and external search loop (the user feels a symptom and actively queries a search engine) [36]. Wearable technology shifts this dynamic into a passive, continuous, and internal loop. The device acts as an automated external trigger, delivering instant haptic or visual ‘digital alarms’ (e.g., sudden heart rate warnings or drops in readiness scores) that intercept the user’s cognitive bandwidth without their explicit initiation.

Phenomenologically, this manifestation of constant algorithmic feedback seeking demonstrates notable neurobehavioral similarities with recognized patterns of behavioral and digital addictions [37,38]. The continuous stream of biometric data operates via intermittent reinforcement schedules; the user compulsively checks the device anticipating a ‘normal’ validation score, which yields a transient state of anxiety relief that quickly decays, thereby reinforcing the subsequent checking cycle [3]. Over time, a tolerance-like phenomenon develops, whereby the individual requires increasingly frequent algorithmic reassurance to mitigate escalating somatic anxiety. Consequently, pre-existing cyberchondria provides the latent anxious framework, but the relentless telemetry of the wearable serves as the accelerative catalyst, transitioning the individual into a state of techno-hypochondria where algorithmic authority completely supersedes subjective bodily sensations.

As shown in Table 3, contrasts with cyberchondria must not oversimplify the existing literature. While cyberchondria frameworks recognize that online information-seeking can be triggered by wearable alerts [26] traditional cyberchondria severity scales (CSS) remain limited to user-initiated search behaviors rather than continuous, automated biometric loops. Similarly, while Etkin beautifully argues that personal quantification has “hidden costs” including decreased well-bein [3], Technohypochondria specifically operationalizes this cost within health anxiety. Finally, while showcasing phenotypic similarities to Illness Anxiety Disorder (DSM-5), Technohypochondria is distinct because the anxiety loop is artificially sustained by algorithmic authority rather than internal somatic triggers.

Within the context of clinical practice, the impact of Technohypochondria on healthcare professionals is of a dimension that cannot be ignored. Alzghaibi and Barac et al. demonstrate that nurses and other healthcare workers are also at risk of entering this digital anxiety cycle; indeed, high levels of medical knowledge can paradoxically increase the tendency to theoretically “catastrophize” neutral data from devices [11,25]. It must be noted, however, that while Alzghaibi examines nurses’ perspectives on AI wearables and Barac et al. review burnout detection, neither study directly measured psychometric catastrophizing; hence, these citations serve as analogous support rather than direct empirical proof. This creates new responsibilities for healthcare managers regarding both the preservation of employee well-being and the management of the “misinterpreted data load” coming from patients. Studies conducted specifically within the Turkish sample support that health anxiety is changing form by shifting to digital platforms, and that nurses must assume the role of “digital health coaching” in this process [35,36,37,38,39]. Crucially, while Uzun & Zencir and Arslantaş et al. validate and examine the correlates of cyberchondria in Turkey, their data focus on generalized cyberchondria rather than wearable-focused technohypochondria, serving as a vital contextual proxy for regional digital shifts [35,36,37,38,39].

In light of all these defining attributes and field reflections, our study defines Technohypochondria as: “the individual’s excessive monitoring of biometric data obtained from wearable devices by detaching it from a clinical basis, perceiving normal fluctuations in this data as symptoms of a serious illness and experiencing anxiety, and constantly requiring a validation score from the device to be convinced of their own health status.”

In conclusion, Technohypochondria is a unique clinical manifestation that transcends the traditional information-seeking behavior of cyberchondria and is based on biometric data and algorithmic authority. Our analysis reveals that clarifying this concept is of vital importance for developing digital health literacy strategies in nursing management and reducing technology-induced “cognitive load.” In the 2020–2027 health vision where AI-supported monitoring systems have become standard [40], recognizing and managing Technohypochondria has become an inevitable necessity for the sustainability of care quality. As a theoretical concept analysis, the primary limitation of this discussion remains the current absence of direct psychometric tools to capture these specific metrics, a gap that future field research must actively bridge.

## 8. Identifying Consequences

The outcomes of the Technohypochondria phenomenon on nursing management, patient care, and staff health involve multidimensional and critical risks within the digitalizing healthcare ecosystem. The most concrete reflection of this process manifests as a potential loss of clinical performance and cognitive focus. The concentration of a healthcare professional’s attention on digital data from their own wearable device, rather than on the patient, may lead to disruptions in care processes and theoretically jeopardize patient safety. When technology ceases to be a supportive element and transforms into a constant source of anxiety, it can trigger computational paralysis and technostress, hypothetically leading to a deepening of professional burnout among staff.

From a managerial perspective, this situation presents itself as a potential waste of institutional resources and a loss of workforce productivity. The disruption of human resources planning and work schedules occurs when personnel frequently miss work or seek unnecessary medical tests due to personal health anxieties triggered by device notifications.

As a cumulative result of these processes, the nurse’s ability to make decisions based on professional knowledge and experience can fall under the pressure of numerical data calculated by smart devices. This undermines the nurse’s confidence in their own expertise, making them dependent on validations from the device and leading to a possible weakening of professional autonomy. Prioritizing device-provided scores over the nurse’s own observations in the clinical decision-making process is regarded as one of the newest digital risks facing the modern nursing profession.

## 9. Strengths and Limitations

This study possesses significant strengths in terms of defining a phenomenon that has not yet been clearly named in the literature within the rapidly digitalizing healthcare world and establishing it on a methodological ground. The primary strength of the study lies in its original definition of the “Technohypochondria” concept by isolating health anxiety—which is generally examined under the broad umbrella of “cyberchondria”—specifically within the context of wearable technologies and biometric data tracking. Furthermore, the fact that this analysis is fully aligned with the World Health Organization’s 2020–2027 health vision, where artificial intelligence and digital biomarkers have become standard, ensures its status as a contemporary and guiding resource for nursing management. Methodologically, the rigorous adherence to Walker and Avant’s eight-stage concept analysis model has allowed for the evidence-based structuring of the concept’s antecedents and consequences.

However, there are certain limitations inherent in the nature of the study. As this work is a theoretical concept analysis, the current lack of a valid and reliable measurement tool in the literature to assess the field reflections of the concept restricts the support of the findings with quantitative data. Due to the absence of specific research focusing directly on Technohypochondria, the analysis process was largely shaped by data obtained from pioneering studies in related fields such as cyberchondria and technostress. Finally, the high pace of change in wearable technologies and algorithmic systems may necessitate the future updating of the concept’s boundaries in light of emerging technological features. Building upon this, the analysis is grounded in a synthesis of 35 international studies, which establishes Technohypochondria as a global phenomenon. While regional studies are included to provide empirical depth and demonstrate the cross-contextual consistency of the construct, the proposed framework is intended to serve as a universal theoretical model. It is acknowledged that national differences in digital health literacy and healthcare policies may modulate the expression of this concept, and it is proposed that this model provides a foundational structure that invites further cross-cultural research to confirm its applicability across diverse healthcare ecosystems.

Regarding the methodology, this concept analysis followed the eight-stage Walker and Avant framework, which served as a systematic audit trail to ensure consistency and minimize subjective bias. Throughout the analysis, an iterative process was employed: the conceptual attributes, antecedents, and consequences were continuously refined by cross-referencing against the synthesized literature from 35 international studies. This methodological rigor, combined with a grounding in established nursing management literature, ensured that the resulting framework was driven by empirical evidence rather than idiosyncratic interpretation. The author acknowledges that while formal peer debriefing was not part of this specific workflow, the analysis remained strictly faithful to the structural components of the chosen model, thereby preserving theoretical neutrality throughout the concept structuring process.

The scope of this analysis was limited to English-language sources. While this decision was driven by the necessity to maintain consistency across international databases and to align with the primary language of global digital health research, the potential for language-based bias is acknowledged. The exclusion of literature in other languages—particularly those from non-Anglophone healthcare systems—is recognized as a limitation of this study. It is proposed that future research should aim to incorporate multilingual literature, as this would further broaden the global applicability and inclusivity of the Technohypochondria construct.

## 10. Practical Implications for Nursing Managers

The phenomenon of Technohypochondria should be recognized by nursing managers as a “new generation workforce risk” within the digitalizing healthcare ecosystem. In this context, the following strategic interventions should be implemented at both organizational and individual levels:

- Digital Competence and Literacy: Training programs must evolve beyond basic device usage into a “Critical Digital Health Literacy” curriculum that covers the clinical limits of biometric data from wearable technologies and the management of “data fatigue.” To operationalize this, the curriculum must explicitly include structured pedagogical modules such as “Biometric Baseline Calibration” and “Algorithmic Variance Disambiguation,” training nurses to systematically distinguish benign device-generated artifacts from true physiological anomalies.
- Clinical Focus Procedures: In critical units such as intensive care, “Digital Focus Protection Protocols” should be established to prevent personal device notifications from disrupting clinical decision-making processes and patient safety (via alarm fatigue). Practically, these protocols should mandate institutional rules like “Device-Free Clinical Windows” during high-acuity tasks (e.g., medication administration, bedside handovers) and the mandatory transition of personal wearables to a customized “Clinical Shift Profile” that mutes non-urgent haptic alerts.
- Performance and Risk Management: In root cause analyses of unexpected clinical errors, digital anxiety or algorithm dependence (technostress) experienced by staff should be examined as a variable; “Digital Well-being” parameters should be considered in performance evaluations. This requires integration of a specific “Technological Interference Vector” into standard institutional Root Cause Analysis (RCA) logs and the inclusion of standardized digital burnout metrics during annual managerial performance reviews.
- Managerial Psychosocial Support: For nurses exhibiting Technohypochondria symptoms, “digital detox” awareness should be fostered within the institution, and leadership support should be provided to enable staff to decouple their somatic awareness from algorithms. Managers can actualize this through structured peer-coaching circles and “Analog Verification Exercises,” where nurses are encouraged to re-validate and trust their subjective physical assessment skills over automated, unverified device scores.
- Legal and Ethical Boundaries for Management: These strategic interventions must strictly respect privacy laws and labor regulations. Under data protection frameworks such as GDPR (EU), HIPAA (USA), and the Personal Data Protection Law (KVKK, Turkey), nursing managers are strictly prohibited from accessing, requesting, or monitoring raw biometric data from an employee’s personal wearable device. Therefore, managerial oversight should never involve individual data surveillance. Instead, performance and risk assessments must focus exclusively on observable workplace behaviors—such as clinical attention, response times, and documented operational fatigue—rather than personal device data. Managerial support should be limited to voluntary, non-invasive programs that promote digital well-being, while fully protecting employee privacy and professional autonomy.

In conclusion, Technohypochondria is not merely an individual anxiety issue in digitalized healthcare systems, but a strategic risk area that must be addressed within the context of the ‘attention economy’ and ‘patient safety’ in nursing services management. In technology integration processes, managers must focus not only on the functioning of systems but also on managing the psychological relationship that personnel establish with these technologies. By shifting from passive compliance to proactive technological stewardship, nursing leadership can effectively safeguard both workforce well-being and clinical care quality within the digital frontier.

## 11. Conclusions

This study provides the first comprehensive, systematic concept analysis of Techno-Hypochondria within the context of nursing management and healthcare work environments. By utilizing the Walker and Avant framework, the core attributes, antecedents, and consequences of this emerging digital anxiety loop have been rigorously mapped and successfully delineated from established constructs like Cyberchondria and DSM-5 Illness Anxiety Disorder. As wearable technology and real-time biometric tracking become increasingly ubiquitous among healthcare professionals, Techno-Hypochondria represents a critical psychosocial risk that can accelerate technostress, digital burnout, and cognitive overload, ultimately threatening the stability of health service delivery.

For nursing leaders and healthcare administrators, recognizing this phenomenon is essential for maintaining workforce resilience in the digital era. Proactive institutional strategies—such as implementing targeted educational programs regarding digital wellness and restructuring workplace guidelines to manage device connectivity during high-acuity shifts—are vital to transforming wearable tools from sources of anxiety into instruments of genuine clinical utility. Ultimately, this conceptual mapping clarifies the theoretical boundaries of Techno-Hypochondria, laying a solid foundation for future empirical research, tool development, and targeted managerial interventions aimed at safeguarding the digital well-being of the nursing workforce.

## Figures and Tables

**Table 1:** Core Defining Attributes of Techno-Hypochondria.

Defining Attribute	Operational Definition	Core Manifestations in Healthcare	Key Supporting References
1. Biometric Data Obsession	Hypersensitivity toward continuous tracking of real-time digital biomarkers, reducing somatic sensations entirely into numerical outputs.	- Constant checking of HRV, pulse, and SpO_2_. - Accepting device data as the absolute health authority. - Chronic self-monitoring loops.	Starcevic & Berle (2013) [20]; Etkin (2016) [3]; Lau et al. (2026) [8]; Elgendi & Markov (2026) [5]
2. Digital Misinterpretation & Catastrophizing	Cognitive distortion of normal physiological fluctuations, treating them as acute medical pathologies fueled by advanced medical knowledge.	- Misinterpreting exertion tachycardia as a cardiac event. - Over-clinicalizing non-contextual data. - Cognitive “digital panopticon” effect.	McElroy & Shevlin (2014) [27]; Starcevic et al. (2020) [28]; Miezah et al. (2026) [23]
3. Constant Algorithmic Feedback Seeking	Complete reliance on automated algorithmic scores to validate internal wellness, displacing natural somatic self-awareness.	- Rejecting feeling well without a high “readiness score.” - Total delegation of health state to device charts. - Algorithmic validation dependence.	Han et al. (2025) [24]; Alzghaibi (2025) [25]; Esmonde (2020) [6]; Luo et al. (2026) [26]

**Table 2:** Proposed Item Domains and Structural Roadmap for the Techno-Hypochondria Scale (THS).

Proposed Domain	Conceptual Alignment	Sample Operational Item Markers (Potential Scale Items)
1. Biometric Surveillance Obsession	Attribute: Biometric Data Obsession	- “I feel compelled to check my smartwatch data (e.g., heart rate, HRV) repeatedly during my clinical shift.” - “I rely more on my wearable device’s numerical data to judge my health than on my own physical sensations.”
2. Algorithmic Catastrophizing	Attribute: Digital Misinterpretation	- “When my wearable device flags a sudden fluctuation in my vitals, I immediately assume I have a serious underlying illness.” - “My clinical knowledge causes me to imagine worst-case medical scenarios when my smartwatch detects abnormal metrics.”
3. Externalized Autonomy Dependence	Attribute: Constant Algorithmic Feedback	- “I cannot feel confident about my physical readiness or clinical performance unless my device’s readiness/stress scores validate it.” - “I tend to assume I will make clinical errors if my wearable application indicates poor recovery or sleep quality.”

**Table 3:** Conceptual Differentiation of Techno-Hypochondria from Related Constructs.

Construct	Primary Stimulus	Dominant Cognitive Behavior	Impact on Healthcare Context	Core Theoretical Distinction
Cyberchondria (Zheng et al., 2021) [36]	Online search engines and health forums.	Active, user-initiated information-seeking and symptom matching.	Increases outpatient consult volume based on web findings.	Captures active web queries; cyberchondria scales (CSS) fail to isolate real-time haptic biometric loops.
Quantification Costs (Etkin, 2016) [3]	Continuous personal metrics (steps, performance charts).	Decreased intrinsic motivation and minor drops in subjective well-being.	Leads to general administrative disengagement or fatigue.	Bounded to productivity and general tracking, not acute health-pathology anxiety.
Illness Anxiety Disorder (DSM-5)	Internal somatic sensations or minor body changes.	Persistent preoccupation with having or acquiring a serious medical illness.	High utilization of medical services across clinical settings.	Independent of digital devices; lacks the automated algorithmic validation loop.
Technohypochondria (Proposed)	Real-time, automated wearable biometric data.	Algorithmic catastrophizing and absolute dependence on device scores.	Immediate cognitive interference on duty, potentially impacting performance.	Stands as a specific technology-induced anxiety loop awaiting direct psychometric scaling.

## Data Availability

The data that support the findings of this study are available from the corresponding author upon reasonable request.

## Supplementary Materials

The following supporting information can be downloaded at: https://www.mdpi.com/article/10.3390/healthcare14131971/s1. Supplementary Table S1: Methodological Quality Appraisal of Included Sources via JBI Framework.
