# Supplementary material for: Techno-Hypochondria: A Concept Analysis of Wearable Technology-Induced Health Anxiety Among Healthcare Professionals—Implications for Nursing Management
Source: Healthcare (Basel). 2026 Jul 2;14(13):1971. doi: 10.3390/healthcare14131971 (PMC13360716; doi:10.3390/healthcare14131971)
Supplement: Supplementary file 1 [file healthcare-14-01971-s001.zip › healthcare-4338392-supplementary.pdf]

Supplementary Table 1: Methodological Quality Appraisal of Included Sources via JBI Framework

ID	Author(s) & Year	Design Type	JBI Checklist Instrument Used	Criteria Met (%)	Quality Category
**1**	Powell & Calderon-Smith (2026)	Bibliometric Review	JBI Critical Appraisal for Text/Opinion	83% (5/6)	High Quality
**2**	Elgendi et al. (2026)	Systematic Review	JBI Critical Appraisal for Systematic Reviews	91% (10/11)	High Quality
**3**	Martino et al. (2026)	Theoretical/Systems Exploration	JBI Critical Appraisal for Text/Opinion	100% (6/6)	High Quality
**4**	Miezah et al. (2026)	Scoping Review	JBI Critical Appraisal for Systematic Reviews	82% (9/11)	High Quality
**5**	Luo et al. (2026)	Two-wave Longitudinal	JBI Critical Appraisal for Cohort Studies	91% (10/11)	High Quality
**6**	Arslantaş et al. (2026)	Cross-sectional	JBI Critical Appraisal for Analytical Cross-Sectional	87% (7/8)	High Quality
**7**	Lau et al. (2026)	Systematic Review & Meta-Analysis	JBI Critical Appraisal for Systematic Reviews	100% (11/11)	High Quality
**8**	Gu et al. (2026)	Theoretical Review	JBI Critical Appraisal for Text/Opinion	83% (5/6)	High Quality
**9**	Barac et al. (2024)	Scoping Review	JBI Critical Appraisal for Systematic Reviews	82% (9/11)	High Quality
**10**	Alzghaibi (2025)	Qualitative Study (Nurses' Perspectives)	JBI Critical Appraisal for Qualitative Research	90% (9/10)	High Quality
**11**	Ahmed et al. (2023)	Scoping Review	JBI Critical Appraisal for Systematic Reviews	82% (9/11)	High Quality

ID	Author(s) & Year	Design Type	JBI Checklist Instrument Used	Criteria Met (%)	Quality Category
**12**	Yuan (2022)	Cross-sectional	JBI Critical Appraisal for Analytical Cross-Sectional	75% (6/8)	Moderate Quality
**13**	Han et al. (2025)	Systematic Review & Meta-Analysis	JBI Critical Appraisal for Systematic Reviews	91% (10/11)	High Quality
**14**	Duplaga et al. (2024)	Cross-sectional Survey	JBI Critical Appraisal for Analytical Cross-Sectional	87% (7/8)	High Quality
**15**	Rosman et al. (2020)	Clinical Observation/Opinion	JBI Critical Appraisal for Text/Opinion	100% (6/6)	High Quality
**16**	Gyulai et al. (2025)	Cross-sectional Study	JBI Critical Appraisal for Analytical Cross-Sectional	75% (6/8)	Moderate Quality
**17**	Babu (2025) [Ref 17]	Theoretical Perspective	JBI Critical Appraisal for Text/Opinion	83% (5/6)	High Quality
**18**	Philippe et al. (2022)	Systematic Meta-Review	JBI Critical Appraisal for Systematic Reviews	91% (10/11)	High Quality
**19**	Starcevic et al. (2020)	Narrative Review/Insights	JBI Critical Appraisal for Text/Opinion	83% (5/6)	High Quality
**20**	Zheng et al. (2021)	Systematic Review	JBI Critical Appraisal for Systematic Reviews	91% (10/11)	High Quality
**21**	Jungmann et al. (2024)	Cohort/Experimental Link	JBI Critical Appraisal for Cohort Studies	82% (9/11)	High Quality
**22**	Nadeem et al. (2022)	Cross-sectional / Metacognitive	JBI Critical Appraisal for Analytical Cross-Sectional	87% (7/8)	High Quality
**23**	Uzun & Zencir (2021)	Validation Study	JBI Critical Appraisal for Analytical Cross-Sectional	87% (7/8)	High Quality

ID	Author(s) & Year	Design Type	JBI Checklist Instrument Used	Criteria Met (%)	Quality Category
**24**	Sansakorn et al. (2024)	Cross-sectional Survey	JBI Critical Appraisal for Analytical Cross-Sectional	75% (6/8)	Moderate Quality
**25**	Menon et al. (2020)	Conceptual Review	JBI Critical Appraisal for Text/Opinion	83% (5/6)	High Quality
**26**	Köse & Murat (2021)	Cross-sectional (Adolescents)	JBI Critical Appraisal for Analytical Cross-Sectional	87% (7/8)	High Quality
**27**	Abu Khait et al. (2023)	Cross-sectional Correlational	JBI Critical Appraisal for Analytical Cross-Sectional	87% (7/8)	High Quality
**28**	Khan & Pandey (2022)	Empirical Investigation	JBI Critical Appraisal for Analytical Cross-Sectional	87% (7/8)	High Quality
**29**	Błachnio et al. (2023)	Cross-sectional Empirical	JBI Critical Appraisal for Analytical Cross-Sectional	87% (7/8)	High Quality
**30**	Zheng et al. (2020)	Moderated Mediation Study	JBI Critical Appraisal for Analytical Cross-Sectional	75% (6/8)	Moderate Quality
**31**	Yalçın et al. (2024)	Empirical Modeling	JBI Critical Appraisal for Analytical Cross-Sectional	87% (7/8)	High Quality
**32**	Babu (2025) [Ref 32]	Perspective Essay	JBI Critical Appraisal for Text/Opinion	83% (5/6)	High Quality
**33**	Esmonde (2020)	Qualitative Interview Study	JBI Critical Appraisal for Qualitative Research	90% (9/10)	High Quality
**34**	Prieto-Avalos et al. (2022)	Technical Review	JBI Critical Appraisal for Text/Opinion	83% (5/6)	High Quality
**35**	Etkin (2016)	Experimental / Mixed Empirical	JBI Critical Appraisal for Cohort/Analytical	91% (10/11)	High Quality
